# Evaluating the Effects of Wheat Cultivar and Extrusion Processing on Nutritional, Health-Promoting, and Antioxidant Properties of Flour

**DOI:** 10.3389/fnut.2022.872589

**Published:** 2022-06-16

**Authors:** Sneh Punia Bangar, Kawaljit Singh Sandhu, Alexandru Rusu, Monica Trif, Sukhvinder Singh Purewal

**Affiliations:** ^1^Department of Food, Nutrition and Packaging Sciences, Clemson University, Clemson, SC, United States; ^2^Department of Food Science and Technology, Maharaja Ranjit Singh Punjab Technical University, Bathinda, India; ^3^Life Science Institute, University of Agricultural Sciences and Veterinary Medicine Cluj-Napoca, Cluj-Napoca, Romania; ^4^Faculty of Animal Science and Biotechnology, University of Agricultural Sciences and Veterinary Medicine Cluj-Napoca, Cluj-Napoca, Romania; ^5^Centre for Innovative Process Engineering (CENTIV) GmbH, Syke, Germany

**Keywords:** wheat, extrusion, total phenolics, antioxidants, redness intensity

## Abstract

Wheat has been considered one of the most important staple foods for thousands of years. It is one of the largest suppliers of calories in the daily diet, which is added to many different products. Wheat is also a good source of health-benefiting antioxidants. This study aims toinvestigate the changes in the antioxidant properties, such as total phenol content, 2,2-diphenyl-1-picrylhydrazyl (DPPH), metal chelating activity, 2,2′-azino-bis (3-ethylbenz-thiazoline-6-sulfonic acid) diammonium salt (ABTS^+^) scavenging activity, and color intensity, during the extrusion processing of six different wheat cultivars. The extrusion factors evaluated were 15% feed moisture and two extrusion temperatures (150 and 180°C). Extrusion processing increased antioxidant activity (DPPH, metal chelating activity, and ABTS^+^ scavenging activity), whereas total flavonoids content and total phenolic content were decreased. The L^*^ values of wheat flours increased significantly (*p* < 0.05) after extrusion at 150 and 180°C, 15% mc. Furthermore, redness was decreased from control wheat cultivars (range: 0.17–0.21) to extrusion at 150°C (range: 0.14–0.17) and 180°C (range: 0.1–0.14). The study suggests that extruded wheat could improve the antioxidant potential in food products.

## Introduction

Processing methods of foods can modulate the polyphenol content of foods in several ways (1). Extrusion of cereal-based products has advantages over other usual processing methods because of low cost, short time, high productivity, versatility, unique product shapes, and energy savings (2). It is a versatile and efficient technology involving high temperature, pressure, and short time among different processing methods. It is used to develop infant foods, snack foods, ready-to-eat breakfast cereals, and pet foods, among other products. Extrusion technology is a new economical processing method; it can achieve protein, starch, and cellulose polymer transformation directly or indirectly in a short time (3–5).

Antioxidants as health-promoting food components have been discussed in nutrition science for several years (6–8). The natural or enriched content of antioxidants is associated with health and disease prevention (9, 10). Phenolic acids are the main antioxidants in cereal grains that seem to have enough potential to be beneficial to health by scavenging free radicals, inhibiting lipid peroxidation, and thus exhibiting anti-cancer activity (11, 12). Free radicals are highly reactive forms that persist during the routine metabolic process in our body (13). They are responsible for cell damage which may ultimately lead to diseases. To counteract the negative effect of free radicals, one should eat an antioxidant-rich diet in a routine dietary chart (14). Wheat has been one of the most important staple foods for thousands of years. It is rich in basic nutrients a source of energy, protein, vitamins, and minerals, and contains important amounts of dietary fibers along with bioactive compounds and antioxidant properties (15). Despite the well-known health benefits, recommendations, labeling, and communication campaigns, the majority of cereal foods are made from refined wheat flour and contain less dietary fiber and other health-promoting compounds when compared to whole grain raw materials. Because of its high gluten content, wheat flour is very suitable for making bread and other bakery products. The use of whole grains wheat flour in extruded products can be an effective alternative for obtaining healthier breakfast cereals. The objective of the present study was to provide a comprehensive summary of the changes in the antioxidant properties, such as total phenol content, DPPH, metal chelating activity, ABTS^+^ scavenging activity, and color intensity during the extrusion processing of different wheat cultivars. Diversity is worthwhile because every wheat variety has something to offer in terms of ingredients, taste, and possible uses.

## Materials and Methods

### Wheat Varieties

For this study, six wheat cultivars (cv) (wheat varieties *viz*. PBW-343, WH-896, WH-1080, PBW-590, WH-283, and WHD-943) were collected from Chaudhary Charan Singh Haryana Agricultural University (CCSHAU), Hisar, Haryana, India. The grain of each variety was cleaned and stored (5^o^C) for further evaluation. All tests were performed in triplicates on a dry weight basis.

### De-husking, Milling, and Extrusion of Wheat

Dehusking of wheat samples was carried out using a rice miller, as reported by Punia and Sandhu (16). In the polishing chamber, hulled wheat (150 g) was transferred, and the polisher was run until the husk was fully removed from the grain. The whole wheat flour was made by grinding de-husked wheat in a Super Mill (Newport Scientific, Australia), conditioning it to 15% feed moisture content (mc), and storing it in polyethylene bags for 12 h. A corotating twin-screw extruder was used for the extrusion (Clextral, BC 21, Firminy, France). The screw diameter, (L/D) ratio and die diameter were adjusted to 25 mm, 16 mm, and 6 mm, respectively. The screw speed (400 rpm) and feed rate (20 kg/h) were kept constant. Wheat flour was extruded at 150 and 180°C with barrel temperatures of 50, 100, 125, and 150°C (for 150°C) and 50, 100, 140, and 180°C (for 180°C). An induction heating belt warmed the terminal section of the barrel while running water cooled the feeding section.

### Antioxidant Activity

#### DPPH Radical Scavenging Activity Assay

A modified version of the method described by Brand-Williams et al. (17) was used to measure 2,2-diphenyl-1-picrylhydrazyl (DPPH) radical scavenging activity. Methanol-containing solution of the free radical DPPH has been used. In brief, 100 mg of ground wheat samples were extracted for 2 h in 1 ml methanol and centrifuged for 10 min at 3,000 rpm. The supernatant (100 μl) was treated with 3.9 ml of a DPPH solution containing 6 x 10^−5^ mol/l. Absorbance (A) at 517 nm was recorded after the solution was stored in a dark place for 30 min at 25^o^C.

The DPPH activity was calculated using the following formula (18, 19):


$$\begin{array}{l} DPPH\;\left(\%\right)=\;\frac{Abs_{DPPH}-Abs_{sol}}{Abs_{DPPH}}\times 100 \end{array}$$

where Abs_DPPH =_ Absorbance of DPPH solution and Abs_sol =_ Absorbance of extracts.

#### Metal Chelating Fe^2+^Activity

The already published method described by Dinis et al. (20) was used to determine metal chelating activity. Briefly, 50 μl of ferrous chloride (2 mM/l) was mixed with 0.5 ml extract, and 1.6 ml of 80% methanol was added. After 5 min, the reaction was started by adding 5 mM/l ferrozines (100 μl) and vortex shaking the mixture. For 10 min, the mixture was incubated at room temperature (25°C). A spectrophotometer (UV-VIS-NIR Spectrophotometer UV-3600 Plus Series, Shimadzu, Kyoto, Japan) was used to measure the absorbance (A) of the solution at 562 nm. The following formulae were used to calculate the extract's Fe^2+^ chelating activity (7):


$$\begin{array}{l} \text{Iron }(\text{F}{\text{e}}^{2+})\text{ chelating activity \% }=\\ \{1-(\text{ A of sample at }562\text{nm}/\text{Absorbance of control at }562\text{nm})\}\text{x }100 \end{array}$$

#### ABTS^+^ Scavenging Activity

Generally known as the ABTS, the 2,2'-azinobis (3-ethylbenzothiazoline-6-sulfonic acid) diammonium salt (Sigma–Aldrich Chemie, Steinheim, Germany) is a spectrophotometric method for determining the antioxidant properties of different compounds. An improved ABTS decolorization test was used in the study (21). The oxidation of ABTS using potassium persulfate (BDH, Poole, UK) generates ABTS^+^. In a disposable microcuvette, 3 ml of ABTS cation solution was combined with 30 μl extract, and the decrease in absorbance was evaluated after 1 min of incubation. Different concentrations of vitamin C were used to create a standard curve. The scavenging ability of ABTS^+^ was measured in μmol of ascorbic acid equivalents (VEAC) per gram of wheat.

### Total Phenolic Content

Total phenolic content (TPC) in studied extracts was evaluated using the already published method described by Gao et al. (22) using the Folin-Ciocalteu- Sigma-Aldrich, Buenos Aires, Argentina reagent. Briefly, 200 mg (wheat flour) were extracted for 2 h at room temperature (25°C) with 4 ml acidified methanol (HCl/methanol/water, 1:80:10, v/v/v) using a wrist action shaker. On a centrifuge, the mixture was centrifuged at 3,000 g for 10 min (REMI, Mumbai, India). The total phenolic content of the supernatant was determined. A 200 μl aliquot of the extract was added to a 1.5 ml Folin–Ciocalteu reagent that had been freshly diluted (10-fold). After allowing the liquid to equilibrate for 5 min, 1.5 ml of sodium carbonate solution (60 g/l) was added. The absorbance of the combination was measured at 725 nm after 90 min of incubation at room temperature (25°C). As a blank, acidified methanol was used. The results were calculated in gallic acid equivalents (GAE)/g of flour. All of the analyses were carried out in duplicate.

### Total Flavonoid Content

Jia et al. (23) previously described the method for determining total flavonoids content, and for this study same method has been used. In brief, 1.25 ml of distilled water was used to dilute the wheat extract (250 μl). The sodium nitrite (75 μl of a 5% solution) was added, and the mixture was allowed to stand for 6 min. In addition, 150 μl of a 10% aluminum chloride solution was added, and the combination was allowed to react for 5 min. Thereafter, the solution was then stirred well with 0.5 ml of 1M sodium hydroxide. A Spectrophotometer-Thermo-scientific was used to detect the absorbance at 510 nm. The standard was catechin, and the results were expressed as g of catechin equivalents (CE)/g of flour.

### Color Characteristics

Color measurement of flours has been analyzed based on the L^*^, a^*^, b^*^ color scheme, and a Hunter Colorimeter with an optical sensor (Hunter Associates Laboratory Inc. Restan VA., USA) was used. The color difference (ΔE) was calculated as:


$$\begin{array}{l} \Delta E={\left\{\left(dL^{*}\right)+\left(da^{*}\right)+\left(db^{*}\right)\right\}}^{1/2} \end{array}$$

Redness intensity (RI = a^*^/b^*^) was calculated for each sample cultivar (PBW-343, WH-896, WH-1080, PBW-590, WH-283, WHD-943).

## Data Analysis

All of the tables' data is an average of triplicate observations that were subjected to one-way ANOVA, applying Minitab statistical software version 14 (Minitab Inc., USA).

## Result and Discussion

### Antioxidant Activity

#### DPPH Radical Scavenging Activity Assay

**Figure 1:**
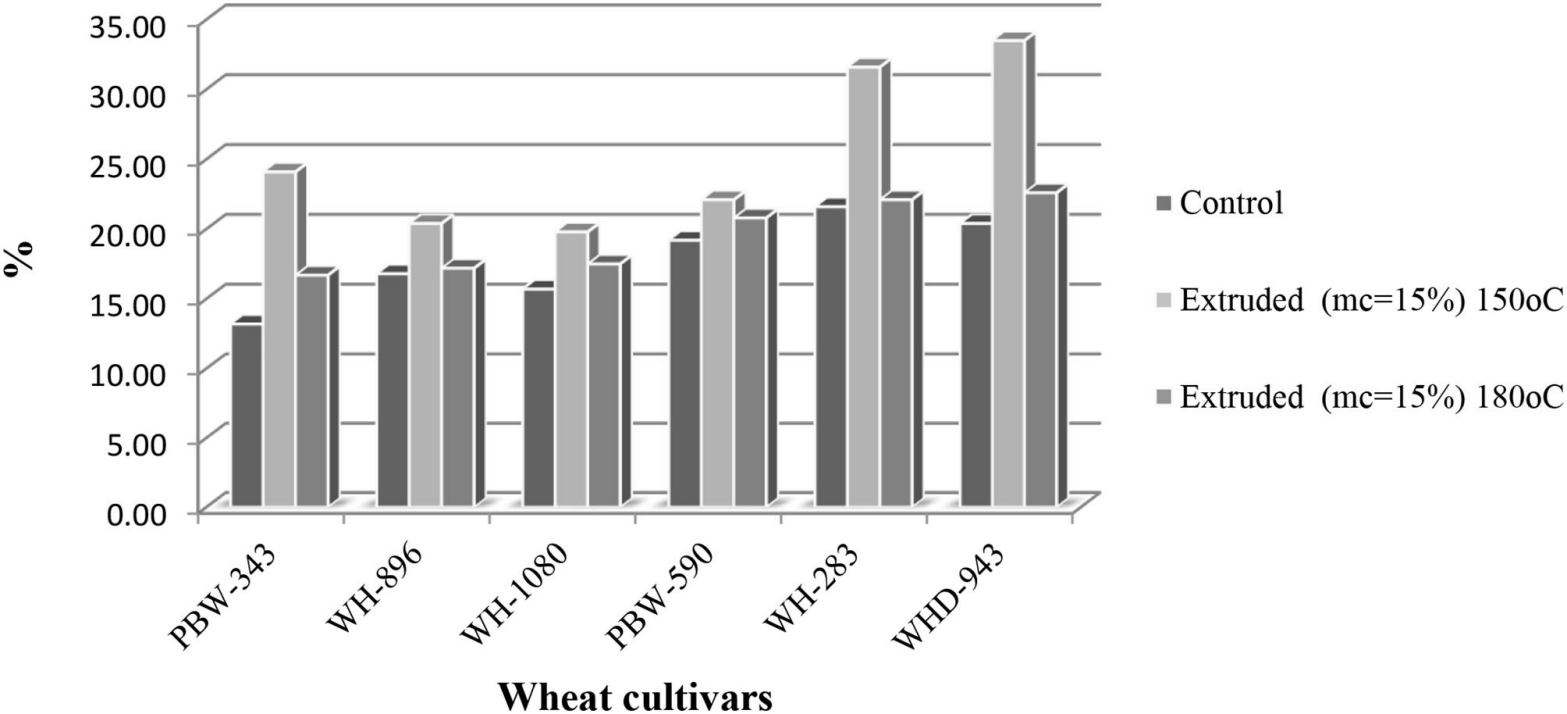
2,2-diphenyl-1-picrylhydrazyl (DPPH) Radical Scavenging Activity Assay (%) of control and extruded wheat cultivars (PBW-343, WH-896, WH-1080, PBW-590, WH-283, and WHD-943) flours at different temperatures (150 and 180°C, mc = 15%).

The DPPH procedure assesses the target compounds' hydrogen donating capacity in a methanolic media. Antioxidative activities of wheat flour samples with extrusion are shown in Figure 1. Percent DPPH inhibition shown by control (non-extruded) wheat cultivars ranged from 13.2 to 21.6%, with cv.WH-283 and cv.PBW-343 shows the highest and the lowest values, respectively. Compared to control wheat samples, extrusion cooking significantly increased DPPH free radical scavenging activity in all wheat cultivars. Antioxidant activity varied significantly among cultivars when the extrusion was carried out at 180°C and 15% mc. The DPPH ranged from 16.7 to 22.6%, with the highest and lowest being cv.WHD-943 and cv. PBW-343, respectively. Also, the DPPH in the extrudates (150°C, 15% mc) varied significantly among cultivars that ranged between 19.8 and 33.5%. The highest and the lowest increase were exhibited by cv. WHD-943 and cv. WH-1080, respectively. Rufian-Henares and Delgado-Andrade (24) and Punia Bangar et al. (7) reported an increase in the antioxidant activity of rye and barley, which could be related to the development of Maillard browning pigments, which boosted the antioxidant activity of extrudates (25, 26).

#### Metal Chelating Fe^2+^Activity

**Figure 2:**
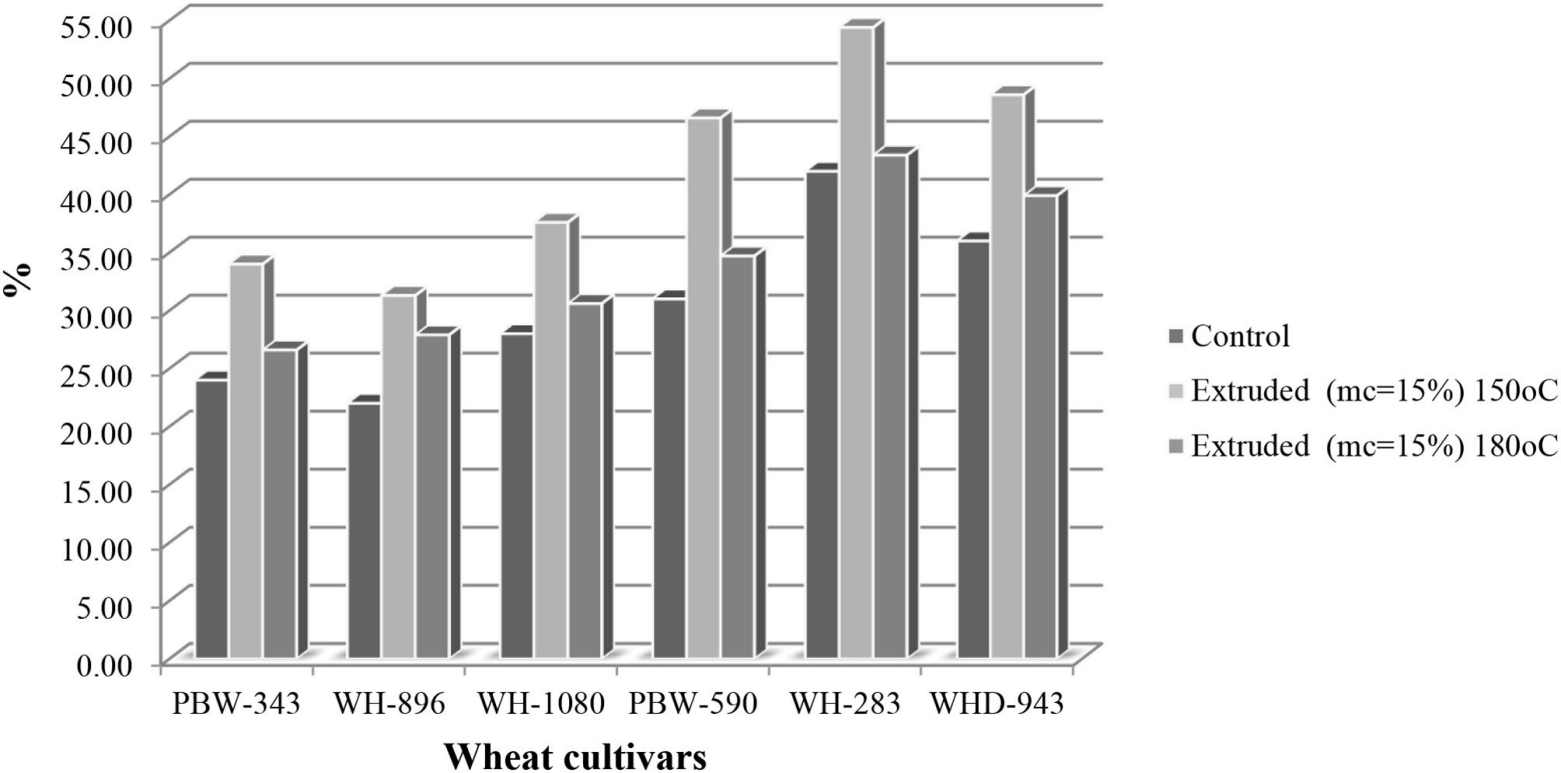
Metal chelating iron (Fe^2+^) activity(%) of control and extruded wheat cultivars (PBW-343, WH-896, WH-1080, PBW-590, WH-283, and WHD-943) flours at different temperature (150 and 180°C, mc = 15%).

Extrusion cooking considerably boosted metal-chelating Fe^2+^activity in all cultivars studied as compared to their corresponding control samples. Metal chelating activity varied substantially (*p* < 0.05) among non-extruded wheat cultivars, ranging from 22 to 42% (Figure 2), with cv.WH-283 and cv.WH-896 having the highest and lowest levels, respectively. When extrusion was carried out at 180°C, and the feed moisture was 15%, the metal chelating activity varied significantly among the cultivars and ranged between 26.6 and 43.4%. The highest and the lowest metal chelating activity was exhibited by cv.WH-283 (43.4%) and cv.PBW-343 (26.6%). When the feed moisture was sustained at 15%, and temperature at 150°C, the metal chelating activity in all wheat cultivars increased and ranged from 31.3 to 54.4%. Cv. WH-283 exhibited the highest, while cv. WH-896 showed the lowest metal chelating power. An increase in the metal chelating activity may be due to the formation of melanoidins during thermal processing (27). Rufian-Henares and Delgado-Andrade (24) reported that the increase in antioxidant activity could be explained by the formation of Maillard browning pigments, which enhanced the antioxidant activity of extruded products.

#### ABTS^+^ Activity

**Figure 3:**
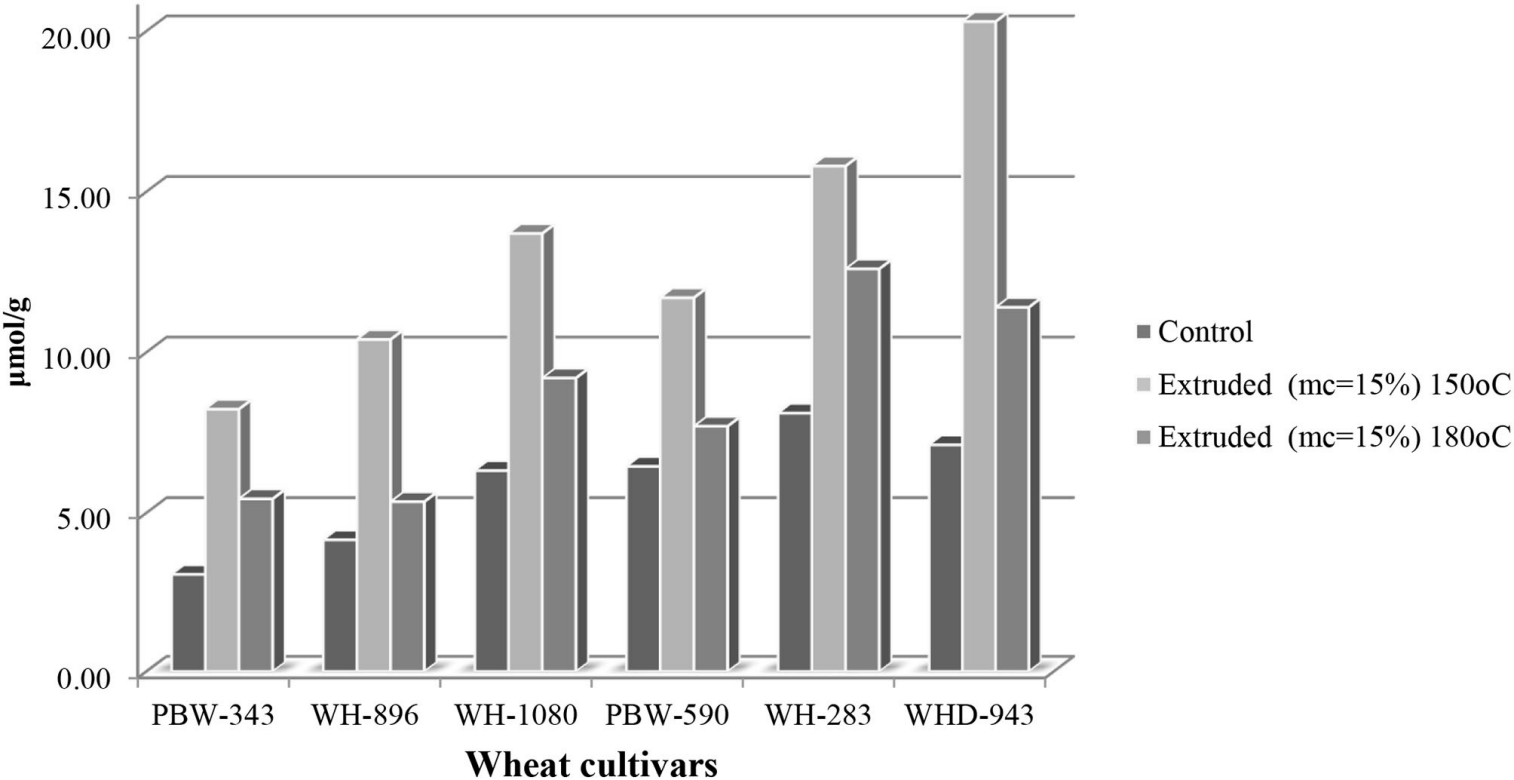
2,2′-azino-bis (3-ethylbenz-thiazoline-6-sulfonic acid) diammonium salt (ABTS^+^) activity (μmol/g) of control and extruded wheat cultivars (PBW-343, WH-896, WH-1080, PBW-590, WH-283, and WHD-943) flours at different temperatures (150 and 180°C, mc = 15%).

The antioxidative capacity of test compounds is assessed by measuring their ability to reduce the ABTS radical anion to its non-radical form. In control wheat cultivars, ABTS scavenging activity ranged from 3.06 to 8.11 μmole VEAC/g, the highest was observed for cv. WH-283 and cv. PBW-343 showed the lowest. Compared to non-extruded samples, extrusion cooking resulted in a considerable increase in ABTS free radical scavenging activity in all cultivars (Figure 3). The ABTS radical scavenging activity in 180^0^C and 15% mc extrudates varied insignificantly (*p* < 0.05) among wheat cultivars and ranged from 5.44 to 12.6 μmol/g, with the highest and lowest being for cv. WH-283 and cv. PBW-343. Further, the ABTS radical scavenging activity in 180°C and 15% mc wheat varieties (PBW-343, WH-896, WH-1080, PBW-590, WH-283, and WHD-943) extrudates varied significantly (*p* < 0.05) among cultivars and ranged from 8.23 to 20.3 μmol/g. The highest and the lowest were observed for cv. WHD-943 and cv. PBW-343, respectively. According to studies (24, 28), pigments (especially melanoidins) are widely known to have antioxidant action. The development of Maillard browning pigments, which boosted the antioxidant activity of extruded goods, could explain the increased antioxidant activity.

### Total Phenolic Content

**Figure 4:**
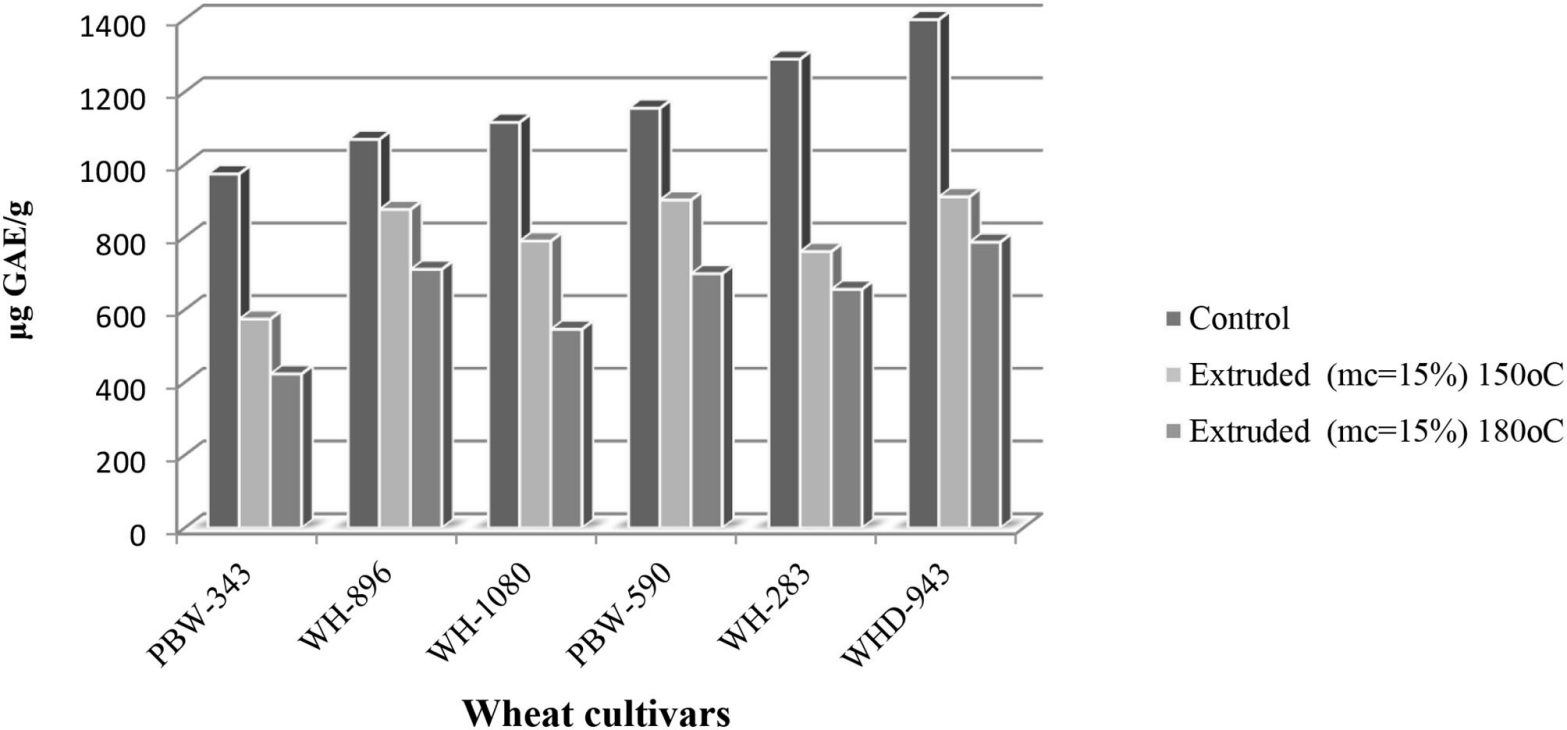
Total phenolic content [TPC (μg GAE /g)] of control and extruded wheat cultivars (PBW-343, WH-896, WH-1080, PBW-590, WH-283, and WHD-943) flours at different temperatures (150 and 180°C, mc = 15%).

The total phenolic content (TPC) in the wheat cultivars ranged from 974 to 1,399 μg GAE/g (Figure 4). Vaher et al. (29) reported TPC of winter and spring wheat varieties ranged from 892 to 569 μg/g. Changes in the TPC can be attributed to differences in wheat cultivars and extraction solvent. When compared to control (non-extruded) wheat samples, the total phenolic content of all cultivars decreased considerably after extrusion. TPC at 180°C and 15% moisture, varied significantly (*p* < 0.05) among cultivars and ranged between 423 to 786 μg GAE/g. The highest and the lowest were observed for cv.WHD-943 and cv.PBW-343. At 150°C and 15%, mc TPC varied significantly (*p* < 0.05) among cultivars and ranged from 524 to 911 μg GAE/g, the highest and the lowest being for cv.WHD-943 and cv.PBW-343, respectively. Sharma et al. (30) reported a decrease in TPC in HTLM (180°C, 15% mc) and LTLM (150°C, 15% mc) extrudates of wheat cultivars. Korus et al. (31) investigated the effect of extrusion on polyphenol content and antioxidant activity of common bean. It has been observed a significant decrease in polyphenol content and antioxidant activity. Repo-Carrasco-Valencia et al. (32) reported that phenolic compounds during extrusion might undergo decarboxylation due to high barrel temperature, and high moisture content may promote polymerization of phenols leading to reduced extractability and antioxidant activity on one side. Thermal treatment induced the hydrolysis of conjugated phenolic compounds resulting in the release of free phenolic acids (33). On the other side, according to Ramos-Enríquez et al. (32), the extrusion process does not cause an increase in the release of TPC due to the addition of methanol during the extraction of TPC. Besides, feed moisture and temperature significantly affect TPC, as already reported (34).

### Total Flavonoid Content

**Figure 5:**
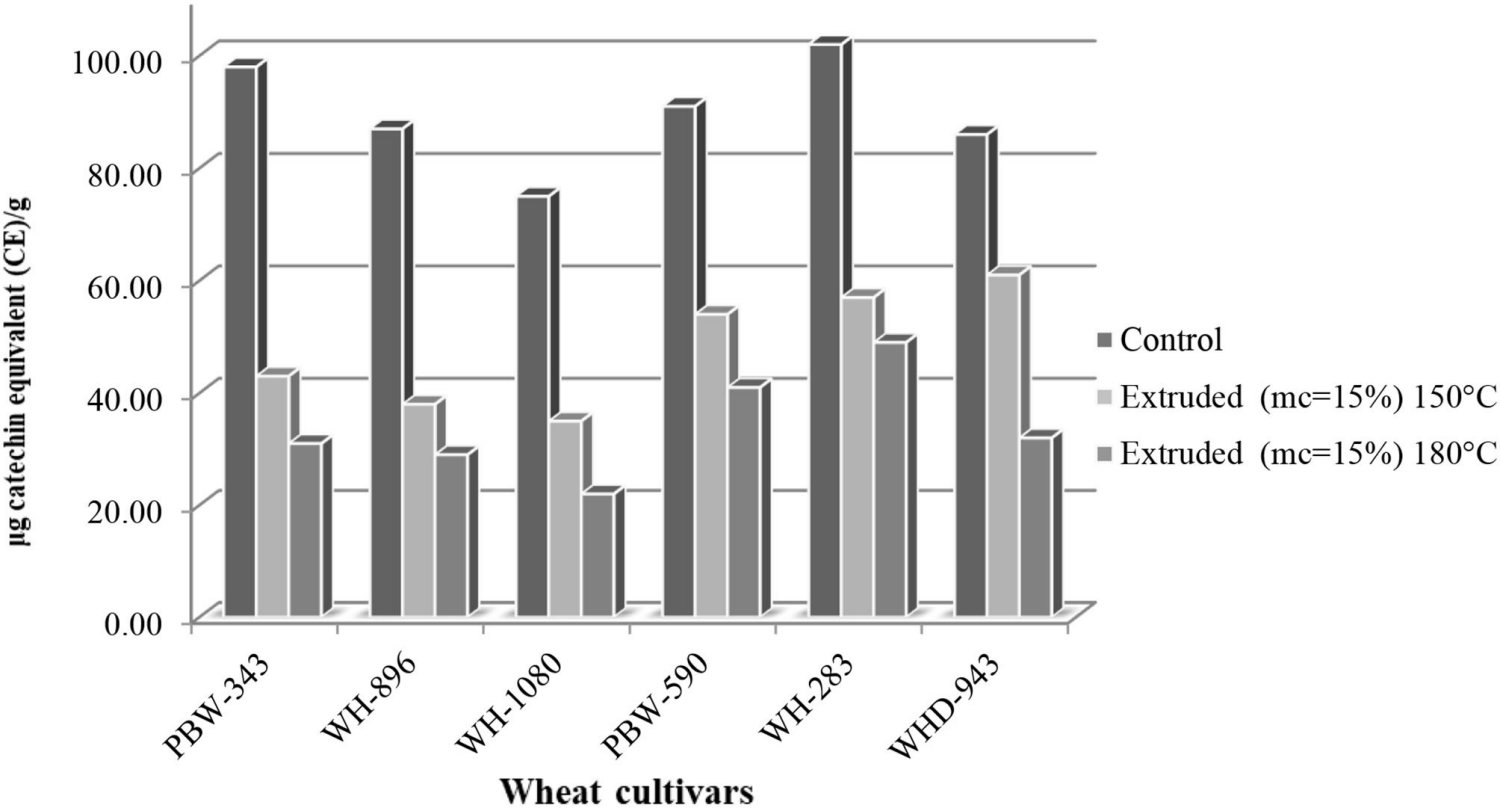
Total flavonoid content [TFC (μg catechin equivalent (CE)/g)] of control and extruded wheat cultivars (PBW-343, WH-896, WH-1080, PBW-590, WH-283, and WHD-943) flours at different temperature (150 and 180°C, mc = 15%).

Flavonoids' antioxidant mechanism may be due to interactions between flavonoids and metal ions, particularly iron and copper (35). The total flavonoid content (TFC) of control wheat ranged from 75 to 102 μg CE/g and varied significantly (*p* < 0.05) among control wheat cultivars (Figure 5), cv. WH-283 had the highest content and cv. WH-1080 has the lowest content. A significant decrease in the TFC was observed during extrusion cooking. TFC levels may have decreased due to the thermal destruction of heat-sensitive flavonoids (36). TFC varied significantly among cultivars in the extrudates (180°C and 15% mc) and ranged from 22 to 49 μg catechin equivalent (CE)/g. TFC varied significantly among wheat cultivars in extrudates (150°C and 15% mc) and ranged from 35 to 61 μg catechin equivalent (CE)/g. as shown in Figure 1. It has also been shown that flavonoids and phenols interact with proteins, causing a loss of their effectiveness (37). Zhu et al. (38) reported that flavonoids are heat susceptible phenolic compounds; therefore, heat exposure during roasting could be a reason for the decrease in TFC.

### Color Characteristics

Hunter Colorimeter was used to analyze the ground wheat extrudates. The lightness is indicated by the L^*^ value, which ranges from 0 to 100. When compared to their equivalent control samples, the lightness of extrudates dropped dramatically. Color characteristics are shown in Table 1. The L^*^ of extrudates at 180^0^C varied significantly (Table 1), and a significant increase in lightness was observed. Cv. PBW-590 and cv. WH-283 exhibited the highest and lowest value of L^*^ (85.3 and 61.4) after extrusion. Extrudates at 150^0^C also showed a significant variation in L^*^ among the cultivars that ranged from 59.8 to 82.8. The moisture content of feed material (wheat flour and brewer's spent grain) had a substantial effect on the L values, also according to Stojceska et al. (39, 40). Extrusion cooking of barley and their blends with tomato pomace resulted in a decrease in lightness, according to Altan et al. (41). The redness (a^*^) varied significantly within the cultivars and ranged from 1.55 to 2.57, and after extrusion, the redness significantly decreased both at 180°C and 150°C. The yellowness (b^*^) of wheat flours from control cultivars varied significantly within the cultivars and ranged from 8.49 to 13.3, and upon extrusion, at 150°C, the b value decreased and ranged between 9.3 and 14.4 and at 180°C the b value increases, the highest for cv. WHD-943 and the lowest for cv. WH-283 was observed.

**Table 1:** Color parameters of extruded and non-extruded wheat cultivars (PBW-343, WH-896, WH-1080, PBW-590, WH-283, and WHD-943) at different temperatures (150 and 180°C, mc = 15%).

**Wheat cultivars**	**Samples**	**L**	a*	b*	RI = a*/b*
**PBW 343**	Control	71.2	1.62	9.31	0.17
	Extruded 150°C	74.3	1.51	9.11	0.16
	Extruded 180°C	79.4	1.34	10.22	0.13
**WH-896**	Control	74.4	2.57	13.2	0.19
	Extruded 150°C	76.9	2.41	14.3	0.17
	Extruded 180°C	82.6	2.02	15.1	0.13
**WH-1080**	Control	75.2	2.08	10.4	0.20
	Extruded 150°C	78	1.91	12.8	0.15
	Extruded 180°C	81.3	1.83	14.6	0.12
**PBW-590**	Control	78.8	2.26	10.6	0.21
	Extruded 150°C	82.8	2.12	12.3	0.17
	Extruded 180°C	85.3	1.98	13.5	0.14
**WH-283**	Control	55.9	1.55	8.49	0.18
	Extruded 150°C	59.8	1.31	9.3	0.14
	Extruded 180°C	61.4	1.20	11.2	0.10
**WHD-943**	Control	75.8	2.62	13.3	0.19
	Extruded 150°C	77.8	2.03	14.4	0.14
	Extruded 180°C	79.6	1.82	16.9	0.10

**Figure 6:**
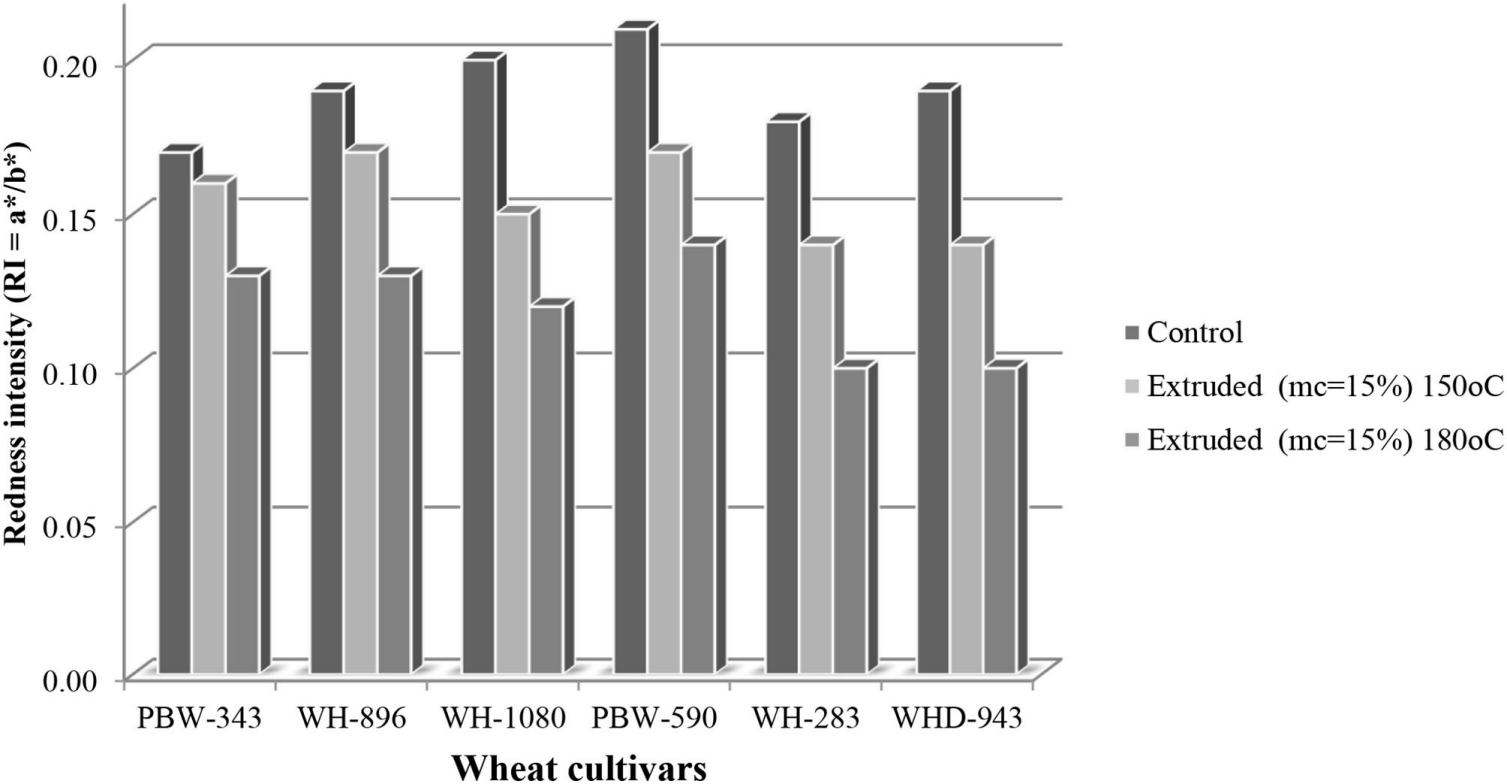
Graphic representation of redness intensity (RI = a*/b*) between control and wheat cultivars (PBW-343, WH-896, WH-1080, PBW-590, WH-283, and WHD-943) at different temperatures (150°C and 180°C, mc = 15%).

*As shown in this Table and Figure 6, RI decreased from control (range: 0.17–0.21) to 150°C (range: 0.14–0.17) to 180°C (range: 0.10–0.14), and differences between wheat cultivars (PBW-343, WH-896, WH-1080, PBW-590, WH-283, WHD-943) are representative*.

## Conclusion

During the present investigation, the effect of extrusion on the antioxidant properties, such as total phenol content, DPPH, metal chelating activity, ABTS^+^ scavenging activity, and color intensity of different wheat cultivars (wheat varieties WH-283, WHD-943, PBW-590, WH-1080, WH-896, and PBW-343), was studied. Results demonstrated that the feed moisture (15%) and extrusion at different temperatures (150 and 180°C) could improve the wheat flour quality. Extrusion increased antioxidant activity (DPPH, metal chelating activity, and ABTS^+^ scavenging activity), compared with total flavonoids content and total phenolic content, which were decreased. Antioxidant-rich flour could be utilized at a commercial scale for the preparation of health-benefiting food/bakery products. An elaborated study could be designed to optimize the processing conditions in this context. Diversity is worthwhile because every wheat variety has something to offer in terms of ingredients, taste, possible uses, and further food and feed applications.

## Data Availability Statement

The original contributions presented in the study are included in the article/supplementary material, further inquiries can be directed to the corresponding authors.

## Author Contributions

All authors participated in the performing, generating, and interpretation of results.

## Funding

This work was supported by a grant from the Romanian National Authority for Scientific Research and Innovation, CNCS—UEFISCDI, project number PN-III-P2-2.1-PED-2019-1723 and PFE 14, within PNCDI III.

## Conflict of Interest

MT was employed by Centre for Innovative Process Engineering CENTIV GmbH. The remaining authors declare that the research was conducted in the absence of any commercial or financial relationships that could be construed as a potential conflict of interest.

## Publisher's Note

All claims expressed in this article are solely those of the authors and do not necessarily represent those of their affiliated organizations, or those of the publisher, the editors and the reviewers. Any product that may be evaluated in this article, or claim that may be made by its manufacturer, is not guaranteed or endorsed by the publisher.
